# Scalable Fabrication of Modified Graphene Nanoplatelets as an Effective Additive for Engine Lubricant Oil

**DOI:** 10.3390/nano10050877

**Published:** 2020-05-01

**Authors:** Duong Duc La, Tuan Ngoc Truong, Thuan Q. Pham, Hoang Tung Vo, Nam The Tran, Tuan Anh Nguyen, Ashok Kumar Nadda, Thanh Tung Nguyen, S. Woong Chang, W. Jin Chung, D. Duc Nguyen

**Affiliations:** 1 Institute of Chemistry and Materials, Nghia Do, Cau Giay, Hanoi 10000, Vietnam; ngoctuan109@gmail.com (T.N.T.); phamquangthuan1982@gmail.com (T.Q.P.); 2 Environmental Institute, Vietnam Maritime University, Haiphong city 180000, Vietnam; tungvh.vmt@vimaru.edu.vn; 3 Advanced Nanomaterial Lab, Applied Nano Technology Jsc., Xuan La, Tay Ho, Hanoi 100000, Vietnam; mark@nanoungdung.vn; 4 Department of Biotechnology and Bioinformatics, Jaypee University of Information Technology, Waknaghat 173215, India; ashok.nadda@juit.ac.in; 5 Institute of Materials Science, Vietnam Academy of Science and Technology, Hanoi 100000, Vietnam; tungnt@ims.vast.ac.vn; 6 Department of Environmental Energy Engineering, Kyonggi University, Suwon 16227, Korea; swchang@kyonggi.ac.kr (S.W.C.); cine23@kyonggi.ac.kr (W.J.C.); 7 Institution of Research and Development, Duy Tan University, Da Nang 550000, Vietnam

**Keywords:** modified graphene nanoplates, graphene additives, antifriction, engine lubricant oil additives, antiwear

## Abstract

The use of nano-additives is widely recognized as a cheap and effective pathway to improve the performance of lubrication by minimizing the energy loss from friction and wear, especially in diesel engines. In this work, a simple and scalable protocol was proposed to fabricate a graphene additive to improve the engine lubricant oil. Graphene nanoplates (GNPs) were obtained by a one-step chemical exfoliation of natural graphite and were successfully modified with a surfactant and an organic compound to obtain a modified GNP additive, that can be facilely dispersed in lubricant oil. The GNPs and modified GNP additive were characterized using scanning electron microscopy, X-ray diffraction, atomic force microscopy, Raman spectroscopy, and Fourier-transform infrared spectroscopy. The prepared GNPs had wrinkled and crumpled structures with a diameter of 10–30 µm and a thickness of less than 15 nm. After modification, the GNP surfaces were uniformly covered with the organic compound. The addition of the modified GNP additive to the engine lubricant oil significantly enhanced the friction and antiwear performance. The highest reduction of 35% was determined for the wear scar diameter with a GNP additive concentration of approximately 0.05%. The mechanism for lubrication enhancement by graphene additives was also briefly discussed.

## 1. Introduction

The worldwide urgency to minimize the effect of greenhouse gases and climate change requires new measures to improve engine efficiency [1]. The freeload and friction losses of diesel engine vehicles account for approximately 10% of the total energy in fuel [2]. The reduction in these losses is crucial for energy efficiency. Techniques such as system design and handling (reducing the size, electrification, and boosting), the addition of systems for the recovery of heating waste, the reduction in friction in the engine, and the improvement in the combustion efficiency have been successfully utilized to improve engine efficiency. In order to theoretically find suitable methods to enhance engine efficiency, the open-source software framework called PERMIX can be employed [3]. Among these techniques, friction reduction has been receiving significant attention from scientists around the world as a key and cost-effective method to maximize the energy efficiency of diesel fuel. One of the major approaches to reducing friction is the use of lubricants, which can be widely applied in automotive, mechanical, and other parts. Lubricants reduce the friction between the interface of two metal parts in relative motion [4]. Additives are commonly added to the blend of lubricants to improve the lubricating efficiency [5,6,7].

Since emerging as a technique to fabricate advanced materials, nanotechnology has provided properties superior to those of traditional bulk materials, and nanomaterials have been intensively used as additives for enhancing lubricant performance. Many nanomaterials such as copper [8], MoS_2_ [9], PbS [10], WS_2_ [11], Zinc borate [12], ZrS_2_ [13], boric acid [14,15], and SiO_2_ [16], have been employed for this purpose. Carbon nanomaterials with many allotropes have remarkable lubricating properties and have also been utilized as additives to improve the performance of lubricant oils. These carbon nanomaterials consist of carbon nanotubes [17,18], porous carbon [19], fullerence [20,21], and graphene [22,23,24].

Graphene, a two-dimensional (2D) carbon material with substantial mechanical, electrical, and thermal properties, has been extensively used in a wide range of industrial applications in the fields of engineering, chemistry, and physics [4,25,26,27,28,29,30]. Additionally, the 2D structures easily slide together, making graphene an effective additive for lowering the friction in mechanical parts and vehicle engines [31,32,33,34]. For example, Zhang et al. successfully fabricated graphene nanosheets from graphene oxides and modified them with oleic acid to be used as additives in lubricant oil to reduce the friction coefficient and wear scar diameter by 17% and 14%, respectively [35]. In another study, Azman et al. blended graphene with 95 vol % synthetic based oil (PAO 10) and 15 vol % palm-oil trimethylolpropane (TMP) ester to reduce the wear scar diameter by 15% [36]. Several works have used graphene as an additive in engine lubricant oil; however, these works either used an expensive graphene-fabricating method (hummer methods) or a low dispersion of graphene in the lubricant oil, which hinder the widespread use of graphene as an additive in the lubricating industry. Furthermore, in order to effectively employ the graphene in practical applications, the dispersion and modification of graphene in any solution are crucial factors.

Herein, we adopt a new and facile method, continuing from our previous work, for the mass production of graphene nanoplatelets (GNPs) by the simple one-spot chemical exfoliation of natural graphite. The resultant GNPs are well-dispersed in water with the assistance of a surfactant. The surfaces of the GNPs are modified to easily and homogeneously disperse the GNPs in the targeted engine lubrication oil with a high stability over a long period of storage time. The prepared and modified GNPs are thoroughly characterized. The enhanced lubricating performance of the GNPs additive-containing lubricant is investigated and discussed.

## 2. Materials and Methods

### 2.1. Materials

Natural graphite flakes were purchased from VNgraphene. Dried acetone, concentrated sulfuric acid (98%), ethanol, sodium dodecyl persulfate (SDS), sodium persulfate (Na_2_S_2_O_8_), and oleic acid were obtained from the Van Minh Company Ltd., Hanoi, Vietnam. The commercial HD-50-based oil was obtained from the petrol station. All chemicals were used as received without further purification.

### 2.2. Synthesis of Graphene Nanoplatelets

The fabricating protocol for graphene nanoplatelets (GNPs) was adopted from our previous work [37]. Natural graphite flakes were added to a 1000-mL reactor containing concentrated sulfuric acid and stirred for 30 min. Sodium persulfate was gradually added into the reaction mixture and further stirred for 3 h at room temperature. The resultant reaction mixture was directly filtered using a glass sintered filter and thoroughly rinsed three times with dry acetone and water to remove any residual reactants. The GNP powder was dried at 60 °C in air and stored for further processing.

### 2.3. GNP Modification

**Figure 1:**
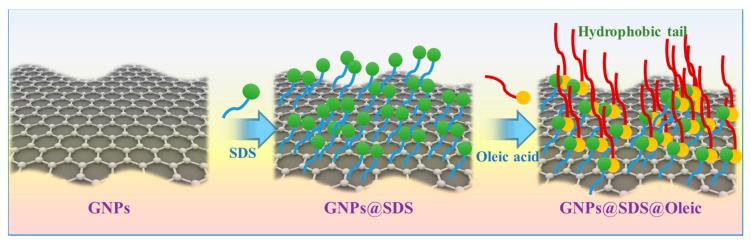
Modification procedure of graphene nanoplatelets with surfactant and organic compound.

Figure 1 illustrates the modification procedures for the graphene nanoplatelets. The GNP powder was first dispersed in an aqueous solution using a combined high shear mixer/probe sonicator system with the assistance of a sodium dodecyl persulfate (SDS) surfactant for 12 h. The homogeneous GNP dispersion in the water with a GNP content of 5% *w*/*w* was used for further modification with oleic acid. For the modification, 15 g of oleic acid was gradually added to 300 mL of GNPs in water with a high shear mixer at 7000 rpm and 80 °C for 3 h. The resultant solution was thoroughly dried at 140 °C, and the oleic acid-modified GNP additive was obtained. The modified GNP additive was added to the HD-50 lubricant base oil with various concentrations ranging from 0.005–0.1% *w*/*w* to evaluate the effectiveness of the additive for enhancing the properties of the lubricant oil.

### 2.4. Characterization

Scanning electron microscopy (SEM), FEI Nova NanoSEM (Hillsboro, OR, USA), was utilized to investigate the morphology of the GNPs obtained from the exfoliation of the graphite flakes. The thickness of the prepared GNPs was measured with an AFM (Bruker Multimode 8 with PF TUNA, CA, USA). Fourier transform infrared (FTIR) measurements were performed on a PerkinElmer D100 spectrometer (Ohio, USA) in attenuated total reflectance mode. Raman spectra were obtained with a PerkinElmer Raman Station 200F (Ohio, USA). Bruker AXS D8 Discover instruments (Texas, USA) with a general area detector diffraction system using Cu Kα source were utilized to obtain X-ray diffraction (XRD) patterns of the prepared samples. A tribological test was performed on the four-ball tribometer (MRS-10A, Shandong, China). The test was carried out at room temperature under a load of 400 N with a speed of 1450 rpm.

## 3. Results

**Figure 2:**
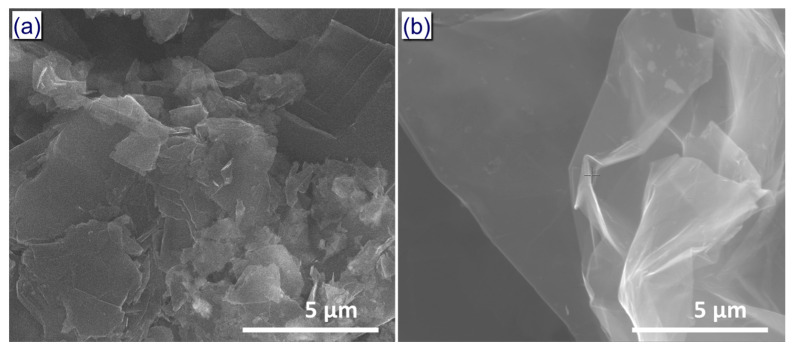
Scanning electron microscopy (SEM) images of (**a**) natural graphite and (**b**) graphene nanoplatelets (GNPs).

The graphene nanoplatelets were facilely fabricated by employing our reportedly improved approach that obtained GNPs from the direct chemical exfoliation of graphite [37]. This method is environment friendly and can be utilized at industrial scale, which is critical for practical applications. The morphology of the natural graphite and prepared GNPs in this work was observed by SEM (Figure 2). The natural graphite flakes have a thick plate structure with dense stacks of graphene layers (Figure 2a). After chemical exfoliation with an oxidant, the graphene layers were detached from a thick plate of graphite flakes, as seen in Figure 2b and Figure S1. The GNPs have a wrinkled structure and a diameter of 10–30 µm. The wrinkled and crumpled morphology indicates that the obtained GNPs consist of a few layers of graphene in each stack, as had been demonstrated previously [38,39]. Additionally, the GNPs’ sheets are semi-transparent to the electron beam (Figure 2b), which is clear evidence that the GNPs contain less than 30 layers of graphene [40,41,42].

**Figure 3:**
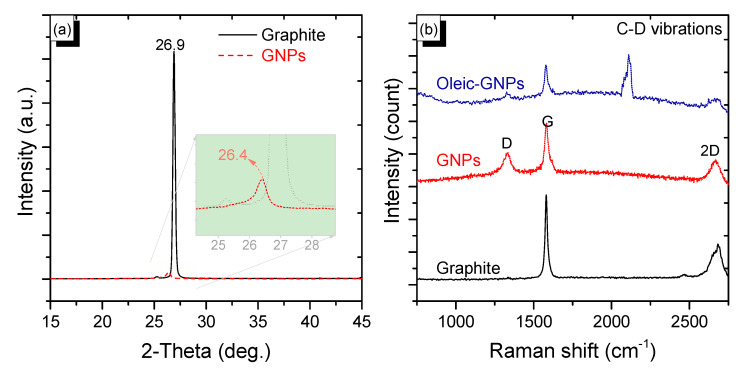
(**a**) X-ray diffraction (XRD) patterns of natural graphite (black line) and graphene nanoplatelets (red line) and (**b**) Raman spectrum of graphite (black curve), graphene nanoplatelets (red curve), and oleic-modified graphenenanoplatelets (GNPs, blue curve).

The crystalline nature of natural graphite and GNPs was determined, and the X-ray-diffraction (XRD) analysis and the results are shown in Figure 3a. The XRD pattern of graphite showed a sharp characteristic peak at 26.9°, which is a 002-diffraction signal [43]. Interestingly, in the XRD pattern of the GNPs, this peak shifts to 26.4° with a significantly broadened and weakened intensity compared to that of graphite, indicating a less orderly structure with multi-layered graphene [37]. This result is consistent with the aforementioned SEM images. The continuous graphene layers as plate structures in natural graphite flakes no longer exist [37]. The multi-layered nature of the resultant GNPs was further investigated using Raman spectrum excited at the wavelength of 633 nm (Figure 3b). The graphite Raman spectrum shows a characteristic G peak at 1580 cm^−1^ and a band at ∼2700 cm^−1^, which belongs to the graphite samples [44]. The Raman spectrum of the GNPs has two characteristic peaks at 1336 (D band) and 1581 cm^−1^ (G band), which correspond to the defects in carbon networks and sp^2^ bonding in carbon elements, respectively [45]. The intensity of the D band peak is significantly lower than that of the G band peak, indicating that the obtained GNPs have fewer defects and a lower oxidant degree when fabricated using this approach. Moreover, the appearance of a peak at 2658 cm^−1^ (assigned to the 2D band), with an intensity significantly lower than that of the G band, indicates that the GNPs are multilayered. The broad photo luminescent band in the Raman spectrum of the GNPs might be due to the amorphous nature of the graphene nanoplatelets. This is consistent with SEM and XRD results. In the Raman spectrum of the oleic-modified GNPs, it can be clearly seen that along with the presence of characteristic bands of GNPs, the CD-stretching vibrations with maximum band intensities around 2100 and 2195 cm^−1^ belong to the oleic acid [46].

**Figure 4:**
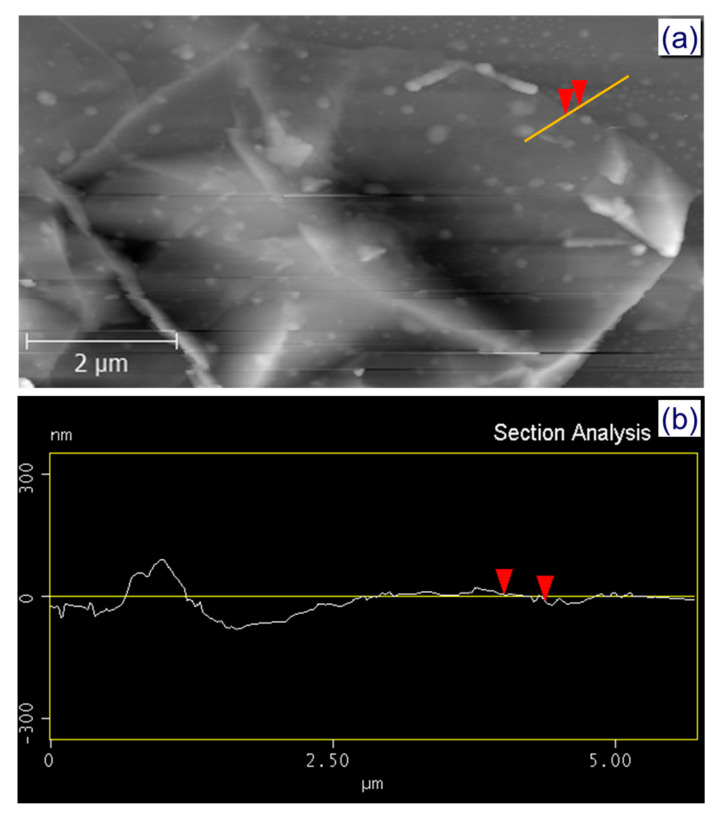
(**a**) Atomic force microscopy (AFM) images of graphene nanoplatelets and (**b**) the height profile calculated from AFM imagery.

The relative thickness of the GNPs can be calculated by atomic force microscopy (AFM) as shown in Figure 4a. The GNPs are not flat because of their crumpled structure that causes the upper graphene layers to protrude from the surface of the Si wafer (Si wafer is substrate to deposit GNPs for AFM measurement) [37]. Figure 4b and Figure S2 exhibit the topographic AFM image of graphene nanoplatelets the height profile derived from the AFM image. The height profile between the GNPs and the Si substrate is utilized to relatively determine the thickness of the GNPs. The average calculated height is approximately 15 nm, which is approximately less than 30 layers considering the gaps between layers. This result is consistent with the aforementioned SEM, Raman, and XRD results on the multilayer nature of the resultant GNPs.

**Figure 5:**
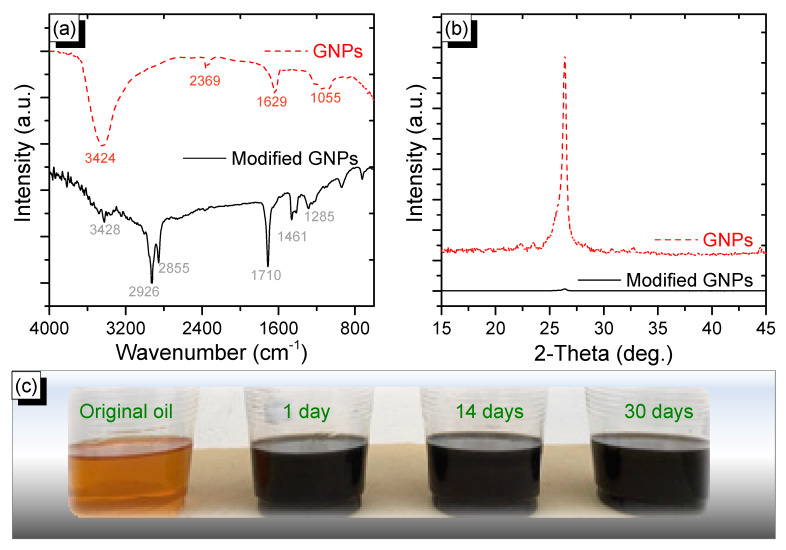
(**a**) Fourier transform infrared (FTIR) spectra, (**b**) X-ray diffraction (XRD) patterns of graphene nanoplatelets (red line) and oleic-modified GNPs (black line), and (**c**) the stability of the oleic-modified GNP additive with 0.01% *w*/*w* in the lubricant oil.

The successful oleic modification of the GNP surface was investigated by FTIR spectra and XRD patterns (Figure 5). In the IR spectrum of the GNPs, the absorption peaks at 3424 and 1629 cm^−1^ are assigned to the vibration band of the –OH stretching group from moisture, which is physically absorbed on the surface of the GNPs [47]. The remaining absorption peaks at 2369 and 1055 cm^−1^ are attributed to the vibration of COO– and C–O stretching, respectively, which could be ascribed to the absorbed CO_2_ [48]. This indicates that the prepared GNPs were virtually not oxidized during the synthesizing process. This is also supported by the X-ray photoelectron spectrometry (XPS) spectrum of C 1s as shown in Figure S3, which shows only one peak of binding energy at 284.5 eV (C–C bonds) indicating that the final product is pure GNPs, and the absence of peaks at 285.5 eV or 286.6 eV is evidence of no oxidizing species. Interestingly, all absorption peaks in the IR spectrum of the GNPs are remarkably weaker, or almost absent, in the IR spectrum of the oleic-modified GNPs, indicating that the surface of the GNPs is uniformly coated by oleic acid. In the IR spectrum of the oleic-modified GNPs, the absorption peaks at 2929 and 2855 cm^−1^ are characteristic of the symmetric and asymmetric vibrations of –CH_2_ (which belongs to the long alkyl chains of oleic acid) stretching, respectively [49]. The sharp absorption peaks at 1710 and 1285 cm^−1^ are assigned to the C=O and C–O stretching vibrations of the carboxylic group, respectively [50]. The band at 1461 cm^−1^ is attributed to the bending vibration of (CH2–) [51]. This result confirms that the entire surface of the GNPs was covered by oleic acid. The bonding between the GNPs and the oleic acid is probably due to π–π interactions [52]. The XRD patterns were further employed to confirm the coverage of oleic acid on the graphene surface (Figure 5b). The characteristic peak at 26.4° for graphene nanoplatelets can be clearly seen in the XRD pattern of the GNPs. After modification with oleic acid, this peak virtually disappeared, indicating that the modified GNP surface is uniformly covered with oleic acid.

The homogeneous dispersion stability of modified GNPs in engine lubricant oil (the oil base is HD 50) was evaluated by observation tests to examine the time period in which graphene can remain in the lubricant oil after the mixing process. The concentration of GNPs in the lubricant oil is 0.01% by weight (Figure 5c). Virtually no sediment is observed after 30 days of storage in static conditions. Additionally, clear straight laser beams were employed to further evaluate the stability of the modified GNPs additive in lubricant oil (Figure S4). The Tyndall effect of lubricant oil with the modified GNPs concentration of 0.01% after one day and 30 days of storage clearly indicate that the modified GNPs additive was well-dispersed in the lubricant oil. Therefore, the modified GNP additive is highly stable in blended engine lubricant oil.

**Figure 6:**
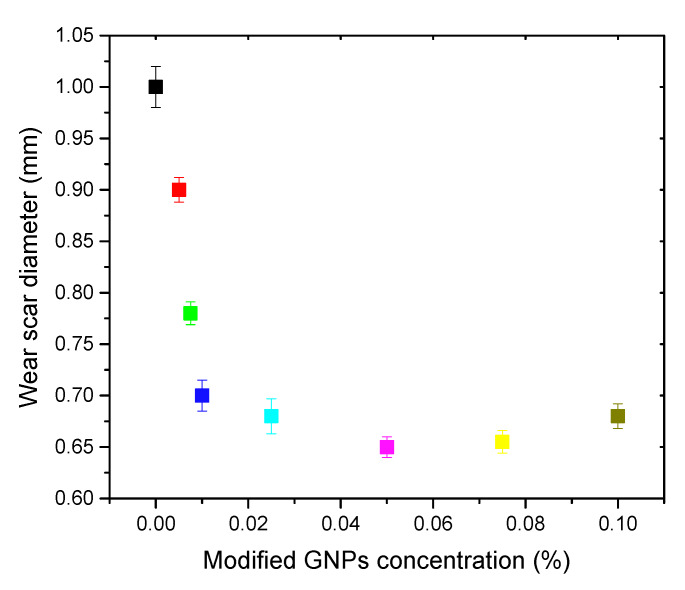
The tribological performance of the engine lubricant oil upon addition of various modified GNPs concentrations.

The wear scar diameter (WSD) is a critical parameter to determine the antiwear performance of lubricant oil. The WSD was evaluated using a four-ball tribometer (MRS-10A, more information about the instrument). The tribological test was performed at room temperature under a load of 400 N and a speed of 1450 rpm. An optical microscope was utilized to measure the diameter of the wear scar on the ball (Figure 6). The WSD is significantly reduced after the addition of the modified GNP additive with a concentration of 0.005% to 0.01% *w*/*w*, indicating that the addition of a small amount of graphene can remarkably enhance the antiwear performance of the lubricant oils. Further increasing the modified GNP concentration from 0.01% to 0.05% *w*/*w* reduced the WSD, which reached a minimum diameter of 0.65 mm with an additive concentration of 0.05% *w*/*w*, representing a 35% reduction in comparison with the WSD using controlled HD-50 base oil. The WSD of the HD-50 with the addition of the oleic-modified GNPs additive is smaller than that of previous works that used graphene as additives for engine lubricant oils (Table 1), most likely caused by better dispersion of the modified graphene in the lubricant oil. An additional increase in the modified GNP content decreases the antiwear properties of the GNP additive for lubricating oil. Thus, the maximal modified GNPs concentration with the highest antiwear properties is approximately 0.05% by weight. Thus, the GNPs additive concentration of less than 0.05% could be selected as the optimal content inside the lubricant oil. The enhanced antiwear performance upon the addition of small amounts of graphene can be explained by the formation of a protective graphene layer on the steel surface. However, when the graphene content increases, the accumulation of the discontinuous graphene film decreases the antiwear properties and causes friction drying [35].

In order to evaluate the thermal stability of lubricant oil upon addition of the modified GNPs additives, the open cup flash points of fabricated oil were determined following ASTM-D92 standard. The results showed that the open cup flash points of the lubricant oil with and without the addition of modified GNPs were 175 °C and 172 °C, respectively, indicating that the GNPs-added lubricant oil was highly stable under the operation condition of diesel oil.

**Figure 7:**
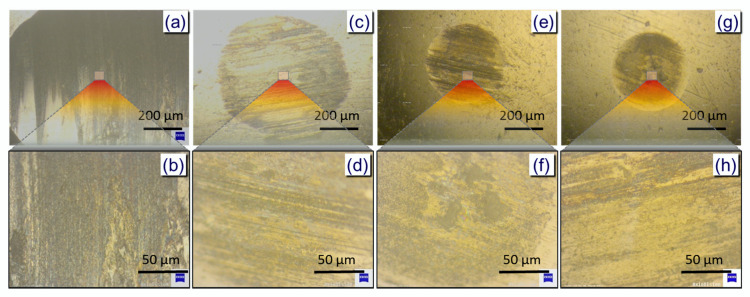
The surface morphologies of the wear scars observed by optical microscopy using different lubricant: (**a**,**b**) base oil, (**c**,**d**) 0.005%, (**e**,**f**) 0.01%, and (**g**,**h**) 0.05%.

The morphologies of the wear scars’ surfaces using lubricants with various modified GNPs contents were investigated by optical microscopy as shown in Figure 7. It can be clearly seen from the figure that when using only base oil, the wear scar is large and the surface is rough with deep narrow trenches. Upon addition of a small amount of the modified GNPs (0.005%), the diameter of the wear scar is reduced and surface becomes smoother, but there still remain deep furrows. However, when the content of the modified GNPs additives was increased to 0.01%, the diameter of the wear scar is significantly reduced to approximately 0.7 mm and the surface becomes much smoother (Figure 7e,f). Further increases in additive contents witness negligible reduction in WSD and smoothness of the wear scar surface. Thus, 0.01 wt % of the modified GNPs additive for lubricant oils was selected as an economically optimized concentration.

In terms of practical application, the price of materials is essential for commercialization. In the market, the average price of graphene nanoplatelets (analytical and industrial grades) ranges from USD 0.6 to 140 per gram (Figure S5). Meanwhile, the GNPs fabricated from the present approach have a price of around USD 1.2 and 15 per gram for the industrial and analytic grades, respectively, including all the expenditures, which is comparative with available commercial GNPs on the market. When it come to the modified GNPs additives for lubricant oils, the determined price of the additive is approximately USD 0.9 per gram. Considering the significant WSD enhancement with only 0.05 w/w % of the GNPs additives in the lubricant oils, the additional cost calculated for 1 kg of lubricant oil is around USD 0.45, which is reasonable in terms of a 35% lubricating enhancement using the prepared GNPs additives. Compared with other available nano-additives for lubricant oils in the literature, the modified GNPs content of 0.05 w/w % is much smaller than that of other nanoparticles (Table 2).

## 4. Conclusions

In conclusion, graphene nanoplatelets were successfully fabricated from natural graphite by direct chemical exfoliation. The resultant GNPs were well-dispersed in an aqueous solution with the assistance of a surfactant and a combination of a high shear mixer and a probe sonicator system. The surface of the graphene was then modified with an organic compound. The as-prepared GNPs were less than 15 nm thick and 10–30 µm in diameter. The results indicate that the modified GNP surface was uniformly covered with oleic acid after modification. The modified GNP additive is facilely dispersed in lubricant oil with remarkable stability, and the GNPs remained in the oil for more than 30 days without settling. The addition of the GNP additive to lubricant oil shows a significant improvement in the tribological performance with a maximal wear scar diameter reduction of 35% at a modified GNP concentration of 0.05% *w*/*w*. The formation of a protective graphene layer on the steel surface is responsible for the enhancement of antiwear performance when using the GNP additive in lubricant oil. This remarkable enhancement of the lubricating efficiency (more than 35% enhancement) uses small amounts of the modified GNP additive (approximately 0.05%) that are cost-effectively fabricated and will diversify the practical applications of graphene in the reduction in energy losses from friction and wear in mechanical processing and automotive components.

## Figures and Tables

**Table 1:** Comparison of the tribological performance between the modified GNPs and those of previous works.

Decreased in Wear Scar Diameter (%)	References
18	[53]
14	[35]
12.6	[32]
Up to 32	[54]
Up to 18.9	[55]
Up to 35	This work

**Table 2:** Optimal concentrations of nano-additives for different lubricant oils.

Nano Additives	Optimum Concentrations, w/w %	References
ZnO	0.5	[56]
CuO	1	[57]
MoS_2_	1	[57]
SiO_2_	0.05–0.5	[16]
Cu-coated carbon	0.5	[58]
ZrO_2_	0.5	[59]
TiO_2_	0.3	[60]
GNPs	0.05	This work

## Supplementary Materials

The following are available online at https://www.mdpi.com/2079-4991/10/5/877/s1, Figure S1: SEM images of prepared graphene nanoplatelets; Figure S2: Topographic AFM image of graphene nanoplatelets and the height profile taken across the white line on the AFM image; Figure S3: XPS spectrum of C 1s; Figure S4: The Tyndall effect of lubricant oil with modified GNPs concentration of 0.01% after 1 day and 30 days; Figure S5: The market price comparisons of graphene nanoplatelets from US, UK, and Chinese companies.
