# Supplementary material for: Scalable Fabrication of Modified Graphene Nanoplatelets as an Effective Additive for Engine Lubricant Oil
Source: Nanomaterials (Basel). 2020 May 1;10(5):877. doi: 10.3390/nano10050877 (PMC7279219; doi:10.3390/nano10050877)
Supplement: Supplementary file 1 [file nanomaterials-10-00877-s001.pdf]

# Supplementary Materials: Scalable Fabrication of Modified Graphene Nanoplatelets as an Effective Additive for Engine Lubricant Oil

**Duong Duc La [1,*], Tuan Ngoc Truong [1], Thuan Q. Pham [1], Hoang Tung Vo [2], Nam The Tran [2,*], Tuan Anh Nguyen [3], Ashok Kumar Nadda [4], Thanh Tung Nguyen [5], S. Woong Chang [6], W. Jin Chung [6] and D. Duc Nguyen [7,*]**

1 Institute of Chemistry and Materials, Nghia Do, Cau Giay, Hanoi 10000, Vietnam; ngoctuan109@gmail.com (T.N.T); phamquangthuan1982@gmail.com (T.Q.P.)
2 Environmental Institute, Vietnam Maritime University, Haiphong city 180000, Vietnam; tungvh.vmt@vimaru.edu.vn
3 Advanced Nanomaterial Lab, Applied Nano Technology Jsc., Xuan La, Tay Ho, Hanoi 100000, Vietnam; mark@nanoungdung.vn
4 Department of Biotechnology and Bioinformatics, Jaypee University of Information Technology, Waknaghat 173215, India; ashok.nadda@juit.ac.in
5 Institute of Materials Science, Vietnam Academy of Science and Technology, Hanoi 100000, Vietnam; tungnt@ims.vast.ac.vn
6 Department of Environmental Energy Engineering, Kyonggi University, Suwon 16227, Korea; swchang@kyonggi.ac.kr (S.W.C.); cine23@kyonggi.ac.kr (W.J.C)
7 Institution of Research and Development, Duy Tan University, Da Nang 550000, Vietnam; nguyendinhduc2@duytan.edu.vn; nguyensyduc@gmail.com (D.D.N.)
* Correspondence: duc.duong.la@gmail.com (D.D.L.); thenam@vimaru.edu.vn (N.T.T.); nguyendinhduc2@duytan.edu.vn or nguyensyduc@gmail.com (D.D.N.); Tel.: +84-966-185368 (D.D.L.)

This provides further information about the SEM images of graphene nanoplatelets. This material is available free of charge via the internet.

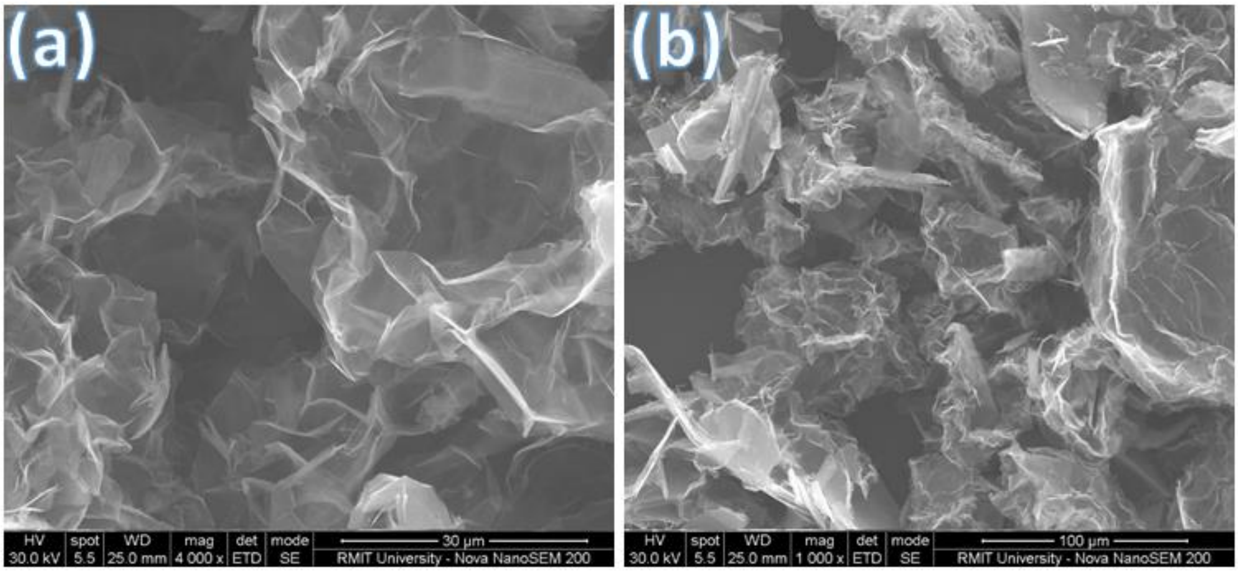


**Figure S1.** SEM images of prepared graphene nanoplatelets.

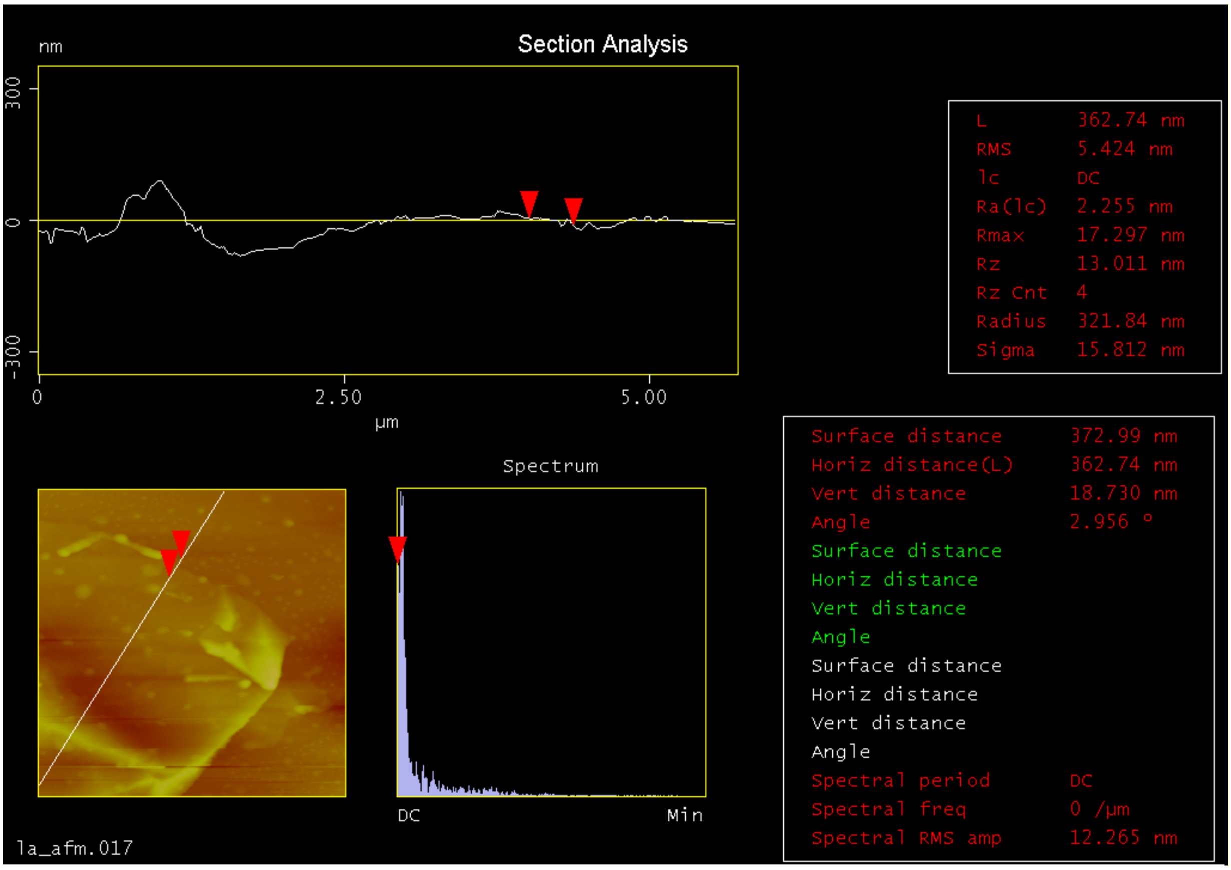


**Figure S2.** Topographic AFM image of graphene nanoplatelets and the height profile taken across the white line on the AFM image.

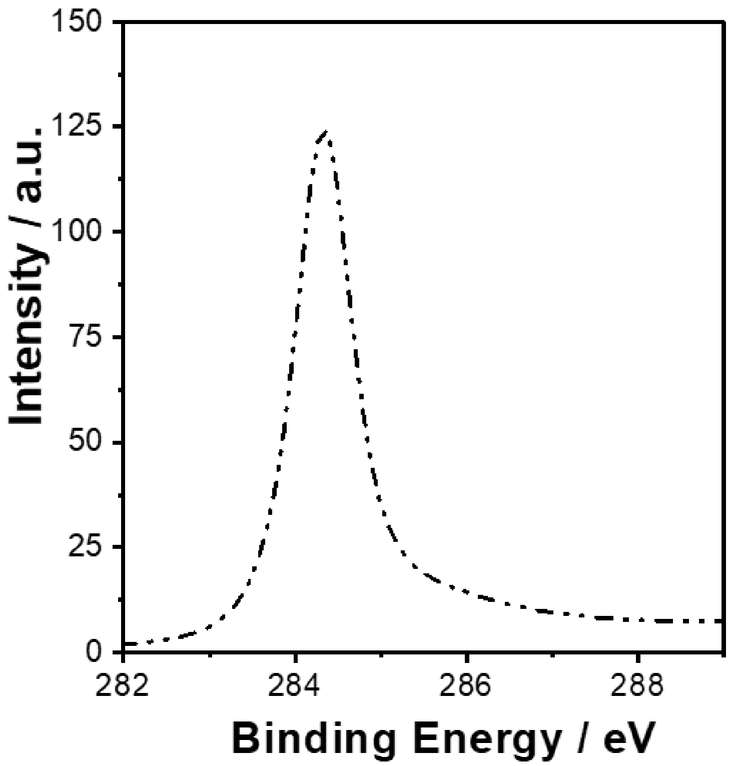


**Figure S3.** XPS spectrum of C 1s.

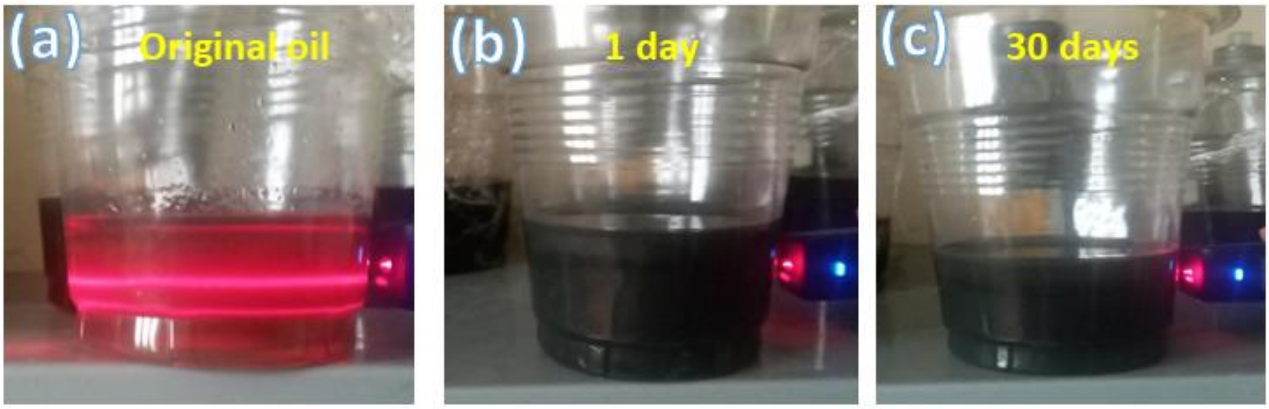


**Figure S4.** The Tyndall effect of lubricant oil with modified GNPs concentration of 0.01% after 1 day and 30 days.

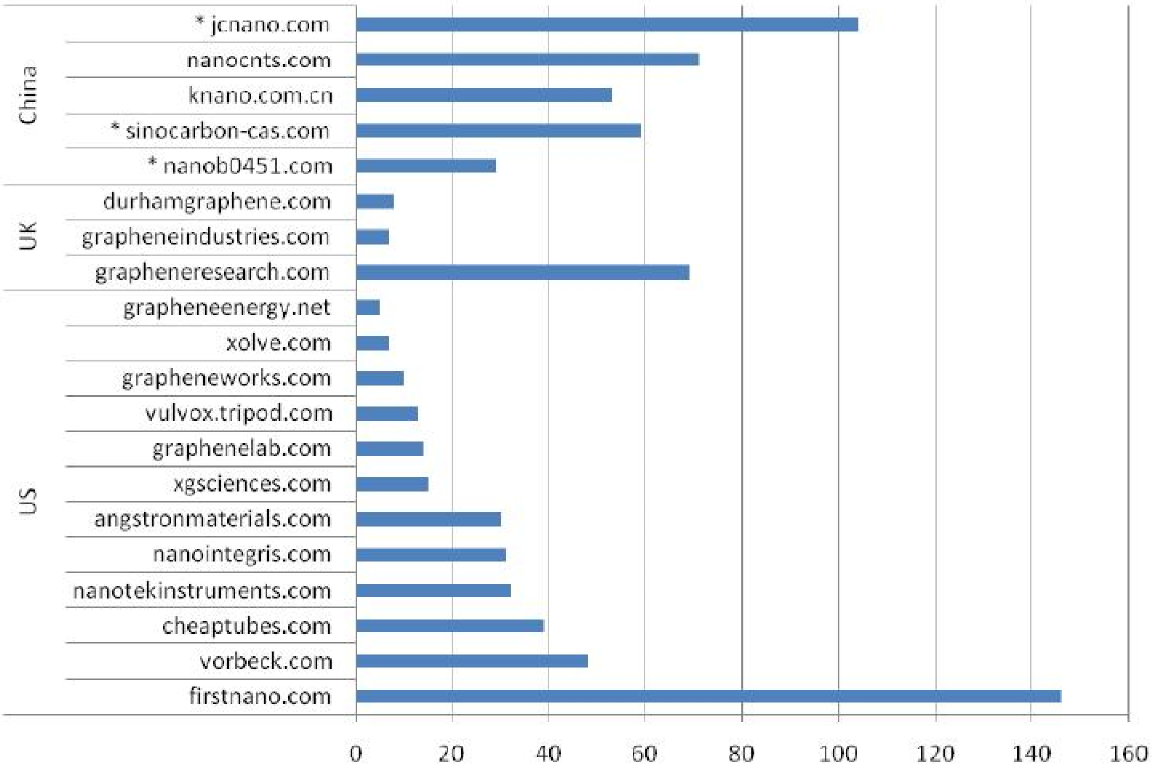


**Figure S5.** The market price comparisons of graphene nanoplatelets from US, UK, and Chinese companies.
